# Non Linear Programming (NLP) Formulation for Quantitative Modeling of Protein Signal Transduction Pathways

**DOI:** 10.1371/journal.pone.0050085

**Published:** 2012-11-30

**Authors:** Alexander Mitsos, Ioannis N. Melas, Melody K. Morris, Julio Saez-Rodriguez, Douglas A. Lauffenburger, Leonidas G. Alexopoulos

**Affiliations:** 1 Dept. of Mechanical Engineering, Massachusetts Institute of Technology, Cambridge, Massachussetts, United States of America; 2 Dept. of Mechanical Engineering, National Technical University of Athens, Zografou, Greece; 3 Dept. of Biological Engineering, Massachusetts Institute of Technology, Cambridge, Massachussetts, United States of America; 4 Center for Cell Decision Processes, Massachusetts Institute of Technology and Harvard Medical School, Boston, Massachusetts, United States of America; 5 European Bioinformatics Institute (EMBL-EBI), Wellcome Trust Genome Campus, Cambridge, United Kingdom; University of Illinois at Urbana-Champaign, United States of America

## Abstract

Modeling of signal transduction pathways plays a major role in understanding cells' function and predicting cellular response. Mathematical formalisms based on a logic formalism are relatively simple but can describe how signals propagate from one protein to the next and have led to the construction of models that simulate the cells response to environmental or other perturbations. Constrained fuzzy logic was recently introduced to train models to cell specific data to result in quantitative pathway models of the specific cellular behavior. There are two major issues in this pathway optimization: i) excessive CPU time requirements and ii) loosely constrained optimization problem due to lack of data with respect to large signaling pathways. Herein, we address both issues: the former by reformulating the pathway optimization as a regular nonlinear optimization problem; and the latter by enhanced algorithms to pre/post-process the signaling network to remove parts that cannot be identified given the experimental conditions. As a case study, we tackle the construction of cell type specific pathways in normal and transformed hepatocytes using medium and large-scale functional phosphoproteomic datasets. The proposed Non Linear Programming (NLP) formulation allows for fast optimization of signaling topologies by combining the versatile nature of logic modeling with state of the art optimization algorithms.

## Introduction

### 1 On Modeling and Optimization

Signaling pathways are of utmost importance for understanding cellular function and predicting cellular response to perturbations [1], [2], [3], [4], [5]. Recent advancements in text mining and the construction of Protein-Protein Interaction (PPI) networks have led to large databases of signaling pathways, showing how proteins interact with each other [6], [7], [8], [9], [10]. However, compilation and visualization of protein connectivity in signaling networks is just the first step towards understanding the cell's signaling mechanisms. The modeling and analysis of these networks either at the connectivity level or down at the level of signal transduction mechanics between nodes is a crucial next step towards the construction of functional models, predictive of the cell's biology.

A variety of methods have been proposed for this task, each adopting a different perspective on the nature of the included reactions [11], [12] and focusing on different properties of the signaling network. Two wide classes of network analysis can be distinguished: i) *Topological analysis* of the signaling network [13], [14], [15] that extracts insight into the cells' function by investigating the structural characteristics of the signaling network (e.g., feedback loops, strongly connected components). ii) *Network identification*, which identifies the network structure (i.e. connectivity of signaling species), or reaction parameters that define the mechanics of signal transduction from one node to the next. Typically a mathematical formalism is adopted to model how signal transduction takes place and an executable model is constructed by combining this formalism with a prior knowledge network (PKN) that serves as a scaffold. By simulating the model under different node and reaction parameters, conclusions can be drawn for the importance of each node and reaction on the propagation of the signal. Amongst the most widely used formalisms are the various forms of logic modeling [16], [17], [18], [19], [20] and ordinary differential equations (ODEs) [21], [22], [23], [24], [25], [26], [27], [28], [29], [30], [31], [34]. In certain cases, the initial model is trained to signaling data via an optimization approach [32], [33] to compute the values of model parameters that better fit the data at hand, or a sensitivity analysis approach is used [35], [36] to compute the influence of model parameters to the overall response of the model. The incorporation of signaling data allows the construction of cell-specific, tissue-specific, or disease-specific pathways [37].

The selection of the modeling approach, and subsequently of the optimization procedure, is very close related to the availability of data and biological question at hand. For example, if time course data are available and the dynamics of signaling reactions are of interest, then an ODE-based approach may be suitable, especially if the interrogated signaling network is small in size. To this end significant work has been published on parameter estimation of ODE-based models using a wide spectrum of methods including general purpose optimization methods (gradient based algorithms, stochastic search algorithms, branch and bound strategies, geometric programming, Dynamic Flux estimation and others)[38]. However, large scale signaling networks cannot easily be addressed within an ODE framework because of excessive CPU times and lack of proper constrain of the association-dissociation constants. If data are available for large pathways but on a single time point, then logic based modeling (Boolean or fuzzy logic, simulated at a ‘pseudo steady-state’) can be used to identify the structure of the signaling pathway.

### 2 Boolean Modeling

In Boolean modeling, signal transduction is modeled using the rules of Boolean logic [39], [40], [41], [42], [43], [44], [45], [46]. Protein nodes assume only binary values {0,1}, denoting the activation (or not) of the corresponding signaling molecule, and signal is propagated from the receptor level to downstream nodes using a combination of OR and AND gates. In [32] an approach was introduced to compress a protein network and convert it into Boolean models that are trained against signaling data. In the approach, implemented in the tool CellNOpt, reactions that appear to contradict the data are removed from the PKN, and thus measurement-prediction mismatch is minimized. In CellNOpt [32] a Genetic Algorithm (GA) was used to prune the pathway by identifying and removing the contradicting reactions. The GA offered a robust and flexible optimization framework and managed to uncover structural differences between normal and cancer liver cell types [23]. In a more recent study, the optimization problem was formulated as an Integer Linear Program (ILP) [5], [47] and was solved through CPLEX (ILOG CPLEX 9.0,)and GUROBI (Gurobi Optimization, Inc., http://www.gurobi.com/)viaGAMS(http://www.gams.com/). In contrast to GA, the ILP formulation guaranteed global optimality and required a fraction of the CPU time needed by the GA. The computational efficiency of the ILP formulation allows the rapid optimization of large scale signaling networks, as illustrated in a study, numbering around 120 nodes and 230 reactions (3 times bigger than the ones interrogated previously) offering a systems wide view of the signaling network in primary human hepatocytes [48].

### 3 Constrained fuzzy logic

Even though Boolean modeling successfully addresses proteins' connectivity and directionality within the signaling pathway, it offers merely a qualitative view of signal transduction. In reality protein activities assume a continuous rather than a 0/1 pattern in signal transduction, making Boolean logic a rough approximation of how signal transduction really takes place. Constrained fuzzy logic (cFL) was introduced to offer a more detailed view of the cell's signaling mechanisms and implemented in the package CellNOpt-cFL[33].

In cFL, a quantitative, yet static view of the signaling network is adopted. Proteins assume real values and a transfer function (TF) is introduced to propagate the signal from one protein to the next [16], [33]. A set of parameters in the TF defines its behavior and allows the calibration of the model to signaling data, in similar fashion to the pruning of the pathway in Boolean modeling. In [33] a two-step method was proposed, wherein first a GA was used to remove all reactions that appear not to be functional based on the data at hand and estimate a rough approximation of transfer function parameters and in a subsequent step, a gradient based/greedy algorithm was used to give a better estimate of the parameters. The cFL approach performed significantly better than Boolean modeling in terms of fitting the data but resulted in more parameters, raising concern about model over-parameterization and causing the training process to be computationally more expensive.

### 4 Proposed approach

Computational efficiency and availability of data are amongst the main limiting factors in modeling via cFL. In the present work we introduce two new approaches for more efficient optimization of signaling pathways in a fuzzy logic framework. Firstly, we formulate the signaling activities as a regular optimization problem (i.e., a nonlinear program (NLP)), solved through IPOPT [49](Interior Point OPTimizer, https://projects.coin-or.org/Ipopt) under GAMS. Secondly, we introduce an aggressive compartmentalization scheme similar to the equivalent classes concept published in [18], to simplify the model at hand so it can be constrained with small datasets. In contrast to previous compression methods, the new compartmentalization procedure is capable of addressing complex connectivity patterns and feedback loops, decreasing in a more efficient manner network size, CPU time, and over-parameterization/non-identifiability caused by the lack of data [50]. As a result, the proposed NLP formulation allows for fast optimization of medium-scale topologies, and can also address the quantitative modeling of large scale signaling pathways. As a case study, we tackle the construction of cell type specific pathways in normal and transformed hepatocytes, to prove that our approach works for pathways as large as 15 receptors wide, numbering around 120 nodes and 230 reactions.

## Results

**Figure 1:**
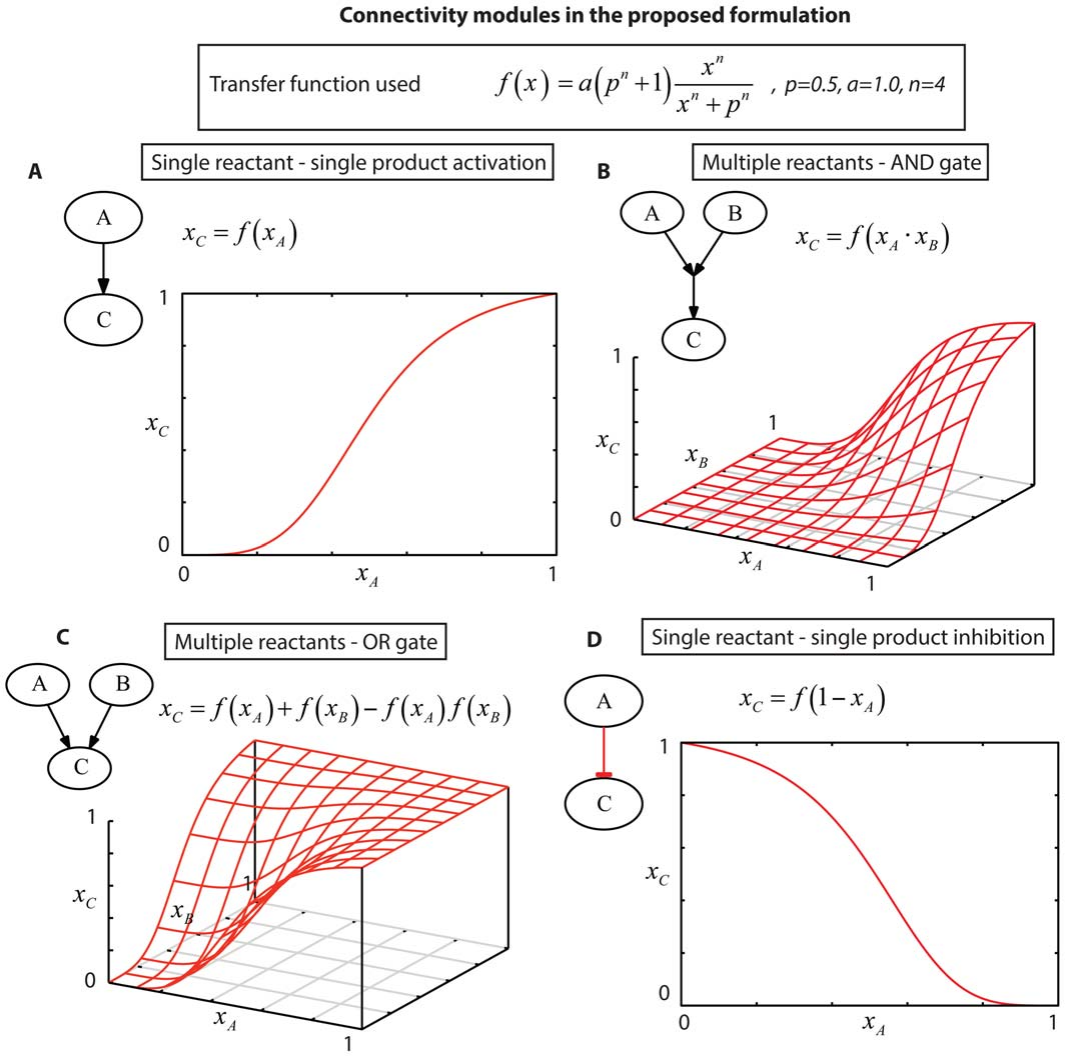
Connectivity modules of signaling pathways in the proposed constrained fuzzy logic formulation. The transfer functions supported by the proposed constrained fuzzy logic (cFL) formulation are illustrated. (A) “single reactant – single product” activation. (B) AND gate with two reacting species. (C) OR gate with two signaling species., (D) “single reactant – single product” inhibition. In all instances, function *f(x)* refers to the normalized hill function, with *p = 0.5, a = 1.0* and *n = 4*.

Our approach is based on the utilization of a transfer function (TF) to model how signal propagates between nodes of the signaling network. Briefly, we implemented and tested the following transfer functions: (i) Unity function 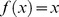, (ii) linear function 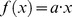 and (iii) normalized Hill function 
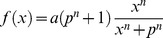
. The normalized Hill function was chosen for being continuous, differentiable, monotonic, and fitting the expected qualitative trends of signaling reactions (sigmoid curve). The normalized Hill function was used in modeling signal transduction in [16], [33]. Reactions with multiple inputs are supported via AND and OR gates. In the case of an AND gate, all of the upstream nodes must be activated for the signal to propagate downstream, while in the case of an OR gate, one of the upstream nodes is enough to activate the downstream node (See Methods section §1). Normalized Hill function, AND and OR gates are shown in figure 1. In this work, we implement an NLP formulation to optimize the value of reaction parameters (*a, p* and *n* for every reaction), minimizing the difference between model predictions and measured data, resulting in a cell-type specific model of the signaling pathway. We then investigate if all reactions were necessary to fit the data by examining the parameters of the reactions and testing to determine if their removal significantly affect model fit.

### 1 Optimization of a Toy Model

**Figure 2:**
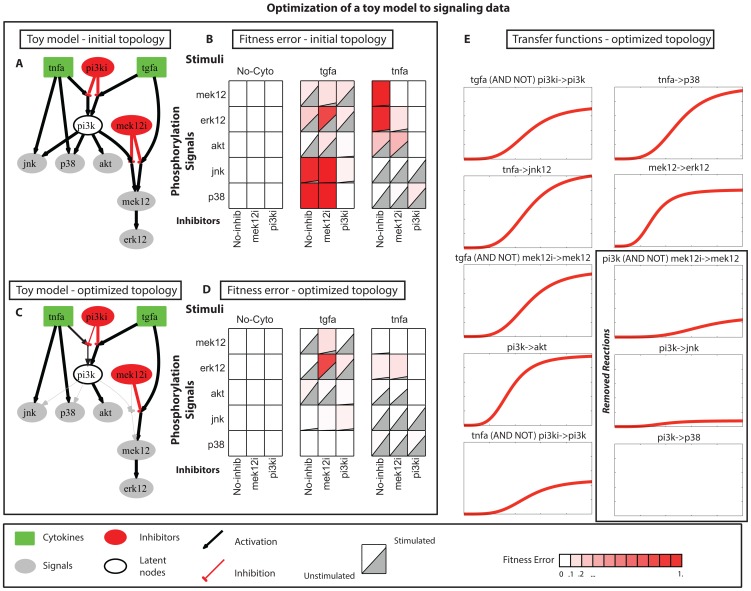
Optimization of a toy model to signaling data. (A) Generic pathway is represented as a signed directed graph, also refers as PKN. Green nodes refer to different cytokines (ligands) where the signaling process initiates; Red nodes refer to inhibitors present in the in-silico dataset; Grey nodes refer to measured proteins; White nodes refer to latent species, i.e. proteins whose activation state is not measured. (B) In-silico signaling data under combinatorial treatment with stimuli (TGFα, TNFα, no-treatment) and inhibitors (mek12i, pi3ki, no-inhibitor). Each subplot shows the average activation level within 30 minutes upon stimulation [37]. Red background refers to model-prediction mismatches (C) Optimized pathway, grey arrows refer to reactions with limited activity (*z_i_^k^*) (caused by *a* parameters being close to 0). The opacity of each edge corresponds to the activity (*z_i_^k^*) of the corresponding reaction. (D) In silico signaling dataset and fitness error after the optimization procedure. Decrease in the red background color shows the optimized model is in accordance to the signaling dataset. (E) Optimized transfer functions presented in C.

To illustrate how the proposed formulation fits parameters *a, p* and *n* to signaling data, we used the 10-node toy model shown in Figure 2A consisting of two stimuli (green nodes); two inhibitors (red nodes); 5 measured signals (gray nodes);4 OR gates (e.g.,TNFα OR PI3K→JNK); 4 AND gates (e.g.,TGFα AND NOT MEK1/2i →MEK1/2); and 4 NOT gates (total number of parameters = 20). *In-silico* data are shown in Figure 2B and consist of 3 stimuli (green nodes); the activation levels of 5 signals (gray nodes); and 2 inhibitors (red nodes) (total number of data points = 45). The red background color in the data (Figure 2B and D) represents the initial and after-optimization measurement-prediction mismatch of the model. For example, MEK1/2 signal under TNFα, without any inhibitor being present, was initially misfitted by the PKN. i.e. The data showed no activation, while in the PKN, MEK1/2 was clearly activated by TNFα. After the optimization procedure the red background was removed, implying that, in the optimized model, TNFα did not activate MEK1/2.

The goal of the NLP formulation is to minimize the fitness error by searching for optimum values of the parameters *a, p* and *n* within predefined bounds. For the toy problem the bounds were: , and while the exponent was held constant n = 4. The upper and lower bounds for *p* were defined in such a manner that p = 0.3 corresponded to an over-responsive transfer function and p = 0.7 corresponded to an under-responsive transfer function, while p = 0.5 was the initial guess for the *p* parameter. Parameter *a* acts as a scaling factor and serves to limit the activity of those reactions that appear not to be functional based on the data at hand. Although the initial selection of upper and lower bounds for the *p* parameters together with the value of *n* was done arbitrarily, in case of high remaining fitness error these values can be updated and the algorithm rerun to guarantee the best possible solution (see also Material and Methods section 5.2 – Definition of search space).

Figures 2C and 2D present the optimization results of the toy model. In Figure 2C, the activity of each reaction is visualized using arrows in gray scale; reactions with larger *a* parameters effectively transmit more signal downstream (are more active) and have a more solid color. The transfer functions themselves are illustrated in Figure 2E. The efficiency of our approach is validated by the eradication of most of the fitness error as shown in Figure 2D (red background). The optimization eliminated the PI3K to JNK, PI3K to P38, and PI3K AND NOT MEK1/2i to MEK1/2 reactions (bottom right panels in Figure 2E). Manual inspection of the data and the initial topology can confirm this decision: JNK and P38 were activated upon TNFα stimulation alone; therefore reactions from the TGFα pathway to JNK and P38 were not active. On the other hand, TNFα stimulation induced AKT activation but did not affect MEK1/2 or ERK1/2, implying that the PI3K to MEK1/2 reaction was not active. To validate that reactions i) PI3K to MEK1/2, ii) PI3K to JNK and iii) PI3K to P38 were not active in the optimized model; we manually removed them from the initial model and run the NLP algorithm once again. No significant differences were observed between the two optimized models, indicating that these three reactions were not vital to fit the data (data not shown).

### 2 Optimization of a medium-scale signal transduction pathway

#### 2.1 Background

**Figure 3:**
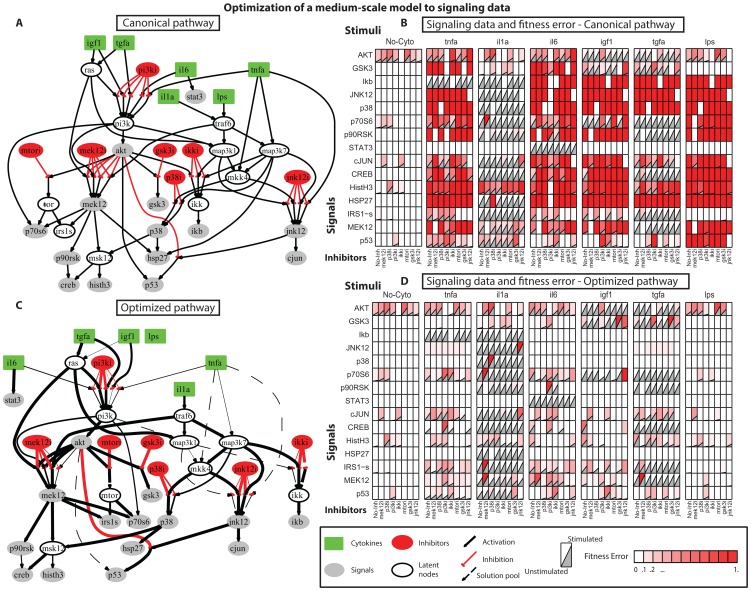
Optimization of a medium-scale model to signaling data. (A) Initial topology as presented in [33]. (B) Signaling data under combinatorial treatments of 6 stimuli (green nodes) and 7 inhibitors (red nodes) reporting 15 signals (grey nodes). The red background represents the measurement – prediction mismatch of the initial topology (46%) (mean fitness error). To generate model predictions, the initial guesses of all model parameters were used (*a = 1.0, p = 0.5*). (C) Optimized pathway. Bold lines refer to the optimized pathway after removing redundant/conflicting reactions. Dashed lines refer to reactions present in the family of solutions that although being redundant are reported since they may bare biological significance. The opacity of each edge corresponds to the activity (*z_i_^k^*) of the corresponding reaction. (D) Signaling dataset and remaining fitness error (8%) (mean fitness error). The red background refers to the fitness error of the solution. Decrease in the red background compared to (B) implies the optimized model successfully fits the signaling dataset (mean fitness error went from 46% to 8%). A and C were generated using graphviz package (http://www.graphviz.org/). B and D were generated using Datarail toolbox [52].

Next, we tested the proposed NLP approach to the medium-scale signaling pathway used in [33], which numbers a total of 52 reactions and 37 species (total number of model parameters = 104). The training dataset was constructed using the xMAP technology on transformed human hepatocytes (HEPG2 cells) [37] and numbers a total of 728 datapoints. The initial topology and the experimental dataset are illustrated in Figure 3A and B. The proposed approach was implemented in 3 steps: (i) definition of search space for the *p* parameters of each reaction, (ii) generation of a family of solutions and (iii) exhaustive removal of reactions from the PKN to address over-parameterization (see Methods section §2, 3, 4).

#### 2.2 Optimization results

Figure 3C contains an “average” pathway for 500 solutions. The solid lines are the minimum set of reactions needed to fit the experimental dataset and the opacity of each of these edges corresponds to the maximum activity (*z_i_^k^*) of the respective reactions. The dashed lines are reactions that were present in the family of models but could be removed for being redundant based on the analysis in Methods section, §4. Figure 3D presents the signaling dataset together with the measurement prediction mismatch for the optimized model (red background). The average CPU time of each run was 10 minutes.

Several interesting features can be uncovered from the proteomic-driven optimization of the generic pathway: LPS pathway was deactivated altogether since it only partially affected the AKT signal. IGF1 and TGFα signaled through PI3K and activated AKT, GSK3 and P70. Moreover, TGFα activated MEK1/2, P90, CREB, IRS1S and HISTH3 via RAS. TNFα and IL1α also had partially overlapping pathways signaling through the MAP3Ks. IL1α signaled through TRAF6 to MAP3K7 and then to JNK, CJUN, P38, HSP27 and IKB. IL1α also activated MEK1/2 via TRAF6 and then P70S6, P90RSK, CREB, IRS1S and HISTH3. TNFα, on the other hand, signaled through MAP3K7 but had clear effects only on IKB, while partially activated a number of signals such as CJUN and P53. Moreover, TNFα partially activated P70S6, CREB, IRS1S and MEK via PI3K.

As shown in Figure 3D, most of the measurement-prediction mismatch has essentially been removed by the optimization procedure. The remaining fitness error is below 8% (mean fitness error). Residual errors appear either in areas of the pathway where the a priori knowledge was poor, or where erroneous measurements in the experimental dataset conflicted each other. The latter is shown in the JNK signal under IL1α and JNKi. Even though JNKi was supposed to have inhibited JNK activation upon IL1α stimulation, the data shows that JNK remained active. In such cases the NLP algorithm is not able to reproduce the respective datapoint. Similar case consisted the misfitting of i) CJUN under IL1α and JNKi, ii) MEK1/2 under IL1α, IL6, TGFα and MEK1/2i, iii) P38 under IL1α and P38i, iv) GSK3 under IGF1, TGFα and GSK3i, and so forth. Those residual errors appeared in almost all optimization procedures [32], [33]. In conclusion, despite the residual error, the optimized model successfully captured the patterns underlying the signaling dataset.

#### 2.3 Cross-validation

**Figure 4:**
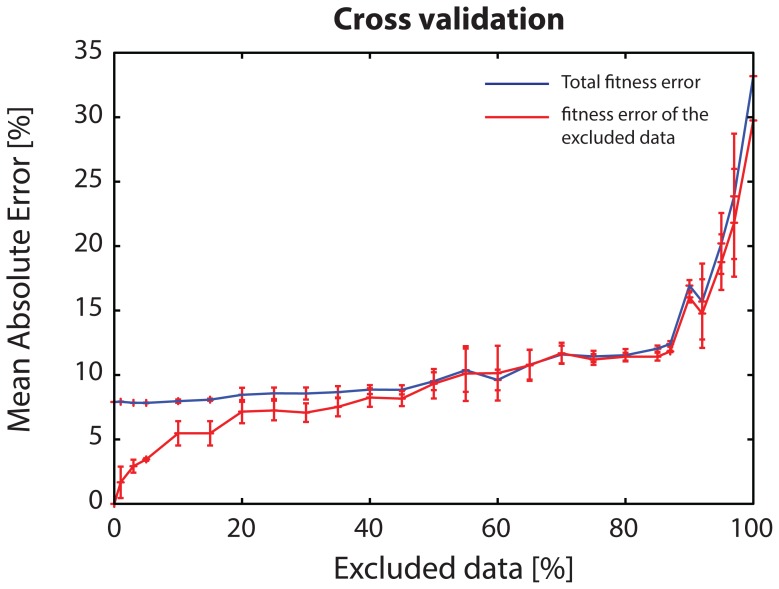
Cross validation of the NLP algorithm (medium-scale pathway). Blue line represents the fitness error corresponding to all measured data (total fitness error); red line corresponds to the fitness error of the predicted (excluded) data. Total fitness error initiates at ∼8% (mean fitness error) and stays relatively stable for excluded portions of the dataset smaller than 40% of the total. Implying that the proposed approach handles efficiently missing data. Even when no data is excluded (0% point in the plot) the total fitness error is at 8% (mean fitness error) because of conflicts in the data or poor prior knowledge of protein connectivity in the PKN. The fitness error corresponding to the excluded data (red line) initiates at 0% since the removal of random portions of the dataset may leave out of the training process datapoints that are easily inferred from the remaining data. E.g. measurement of MEK1/2 under TGFα and IKKi is easily inferred from TGFα and no-inhib experiment. As increasing portions of the data are left out of the training process (excluded data >40%) the fitness error increases significantly. For excluded portions greater than 80% the fitness error quickly reaches that of the null solution.

For the optimization of the PKN, the signaling dataset in its entirety is used. Herein, however, to better evaluate the performance of the proposed formulation, we performed a cross validation study where random portions of the dataset, of increasing size, were left out of the training process, model predictions corresponding to this data were computed and then compared to the measured data evaluating the measurement prediction mismatch. Figure 4 illustrates the fitness error corresponding to all measured data (total fitness error), in blue, together with the error corresponding to the excluded data (in red). Interestingly, up to 40% of the dataset could be removed before the fitness error started increasing significantly, implying the proposed formulation is robust against missing data. Moreover, the algorithm performed relatively well even with 80% of the dataset missing. After this point, a steep increase in the overall error was observed, since key pathways were removed and the fitness error quickly reached that of the null solution.

### 3 Optimization of a large-scale signal transduction pathway

#### 3.1 Background

In order to evaluate the performance of our optimization procedure, we asked whether we could apply the procedure to larger pathways. Here, we focused on pathways that are experimentally identifiable using ELISA type of assays and thus are limited in well-known signal transduction mechanisms. The resultant PKN accounts for dozens of stimuli and their downstream nodes [48]. The pathway contains 228 reactions and 117 species (total number of model parameters = 456). The corresponding data were measured using the xMAP technology on primary human hepatocytes and consist of a total of 120 multi-combinatorial experiments. Cells were perturbed with combinations of 15 stimuli and 3 inhibitors (including the No-inhibitor treatment), while 14 key phosphoproteins were measured (total number of data points = 1680). Before the optimization procedure, the pathway was compartmentalized to reduce the parameters space (the compartmentalized pathway numbers 44 species and 69 reactions, total number of model parameters = 138), while a family of solutions was obtained to guarantee that the algorithm is not trapped in a local minimum (see Methods section §3, 5 and Figure S5).

#### 3.2 Optimization Results

**Figure 5:**
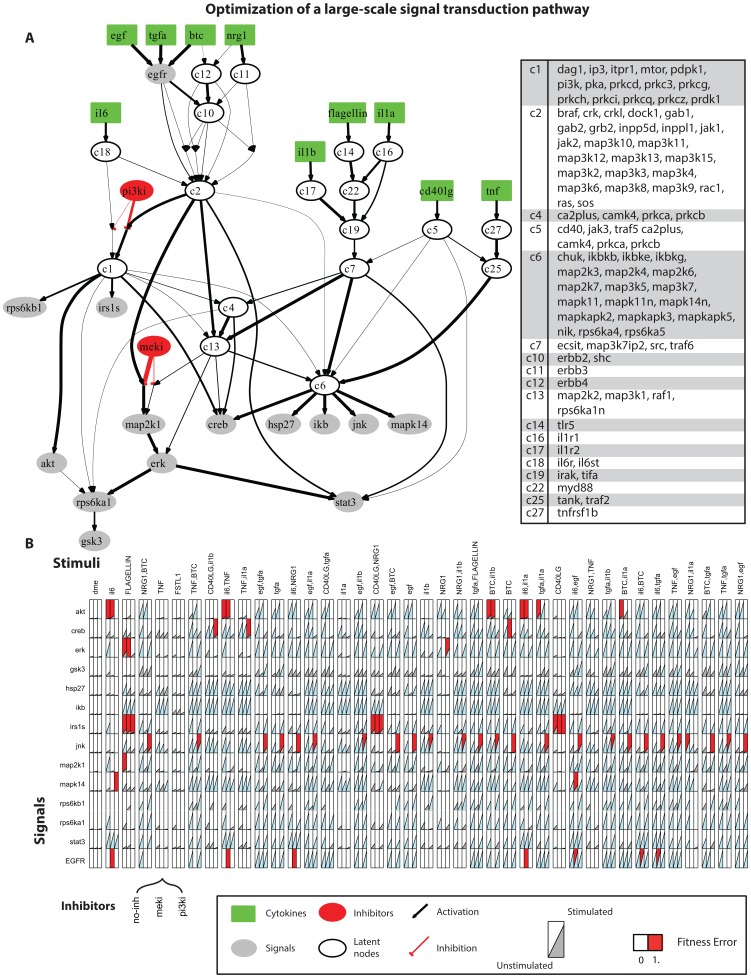
Optimization of a large-scale signal transduction pathway. (A) optimized pathway upon compartmentalization based on the equivalent classes concept (right panel). The proposed compartmentalization scheme groups together nodes that share identical in-silico responses under all experimental conditions, thus decreasing the parameters space. (B) Signaling dataset, consisting of 15 cytokines in combinations of two, and 3 inhibitors (including the no-inhibitor treatment), total of 120 experimental treatments (see [48]). The red background color corresponds to the measurement prediction mismatch of the solution.To generate model predictions the optimized values of all model parameters were used (i.e., parameter values obtained from the optimization procedure)

In Figure 5 the optimized, compartmentalized version of the large-scale pathway is shown, together with the measurement-prediction mismatch. To demonstrate how the compartmentalization scheme works, we first examined the pathways downstream of EGF, TGFα, BTC, NRG1 and IL6. ERBB3 was placed in a group alone (C11) since it was the only node activated by NRG1; ERBB4 was also placed alone (C12) for having been activated by BTC and NRG1. ERBB2 and SHC were grouped together since they were both activated by EGF, TGFα, BTC and NRG1. Moving further downstream, INPP5D, JAK1, JAK2, INPPL1, GRB2, GAB2, GAB1, SOS, RAS, CRK, CRKL, DOCK1, BRAF, RAC1 and the MAP3Ks were grouped into C2 since all of them were activated by EGF, TGFα, BTC, NRG1 and IL6. This example demonstrates how the proposed compartmentalization scheme is based on the experimental treatments present in the dataset. If for example, another ligand was introduced activating via a different pathway RAC1, then the extensive compartment C2 would be broken into 2 smaller ones. First, INPP5D, JAK1, JAK2, INPPL1, GRB2, GAB1, GAB2, SOS, RAS, CRK, CRKL and BRAF, activated by EGF, TGFα, BTC, NRG1 and IL6; and second, RAC1 and the MAP3Ks (MAP3K2,3,4,6,9,10,11,12,13,15), activated by EGF, TGFα, BTC, NRG1, IL6 and the new ligand. With the proposed compartmentalization scheme, the interrogated pathway is never larger than what can be constrained by the data at hand.

**Figure 6:**
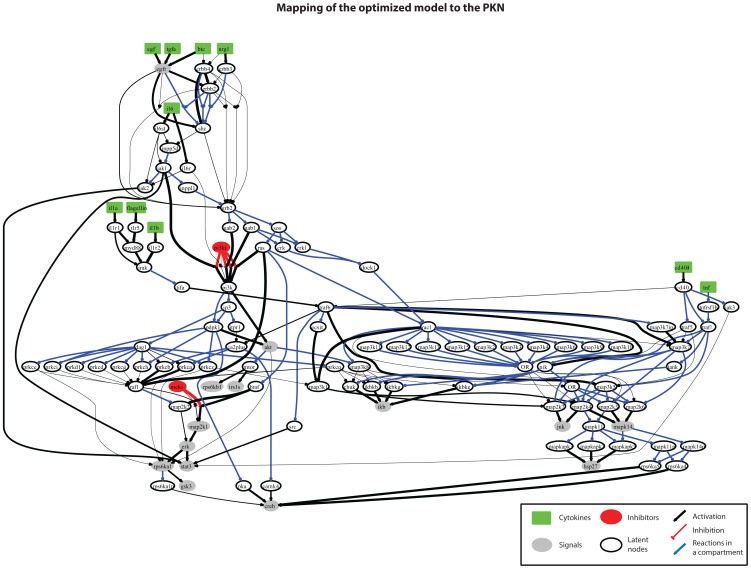
Mapping of the optimized model to the PKN. Mapping of the optimization results to the PKN by removing the compartmentalized components. Reactions within the same compartment are plotted in blue and were not included in the optimization procedure. Reactions in black are the ones whose parameters were interrogated. Their opacity corresponds to their activity in the optimized model, with reactions that propagate more signal downstream being more opaque than the rest.

In Figure 6, the optimized pathway of Figure 5 was mapped back to the PKN. Reactions within the same compartment were plotted in blue and were not involved in the optimization procedure. The rest of the reactions were plotted in black and their thickness corresponds to the maximum activity of each reaction in the optimized model. The resulting pathway reveals well known characteristics of signaling cascades (See [48]): EGF, TGFα, BTC and NRG1, all signaled through the EGFR and then through the cluster of SHC, GRB2, GAB1, SOS, RAS to either activate MAP2K1, ERK, RPS6KA1, GSK3 and STAT3, or go through PI3K to AKT and subsequently to RPS6KB1 and IRS1S. On the other hand IL1β, FLAGELLIN and IL1α signaled through TRAF6 and mainly activated IKB, JNK, MAPK14 and HSP27. CD40LG and TNF activated the same signals but went through TRAF5, TRAF2 and MAP3K7.

The solution obtained herein, when compared to the Boolean solution in [48] was able to decrease the remaining fitness error up to 75% (mean fitness error). The algorithm completed within 20 minutes. Even though the two solutions share the same basic connectivity patterns, the constrained fuzzy logic approach handles conflicts in the data more efficiently, since it allows partial activation of the signaling species. For instance, GSK3 was removed from the Boolean solution for having been activated in an inconsistent manner (it was activated under very few combinatorial treatments and remained unaffected by either PI3Ki or MEKi). Under the constrained fuzzy logic approach, however, GSK3 was activated by RPS6KA1. By fitting the *p* and *a* parameters of this and the upstream reactions the model predictions for GSK3 matched the data and the fitness error was reduced. Similarly, IRS1S and RPS6KB1 were activated under constrained fuzzy logic, in contrast to the Boolean approach.

## Discussion

In this paper we introduced a Non Linear Programming (NLP) formulation for the *quantitative* modeling of signal transduction pathways, based on signaling data. We employed a fuzzy logic approach to model signal transduction mechanisms and coupled it to an NLP optimization formulation. The proposed method allowed for fast optimization of signaling pathways to high throughput signaling data in a quantitative framework. As case study, three pathways of different scale were interrogated, a small, medium and a large-scale one. For the latter two, i) the systematic definition of the search space, ii) the generation of a family of solutions, iii) and the identifiability/over-parameterization of the pathway were addressed to ensure the best possible performance of the proposed formulation. The systematic definition of search space guaranteed that a representative set of solutions was obtained while at the same time minimized the required CPU time. The collection of a family of near optimal solutions decreases the probability of biologically relevant solutions remaining unreported. The proper size for the family of solutions was also addressed (see Supporting Information 1). By addressing over-parameterization either by exhaustively removing reactions from the PKN, or via the proposed compartmentalization scheme, we decreased CPU time and guaranteed that only reactions vital for fitting the data were included in the solution. Finally, results on both the medium and the large scale signaling pathways were compared with the ones obtained by alternative approaches [33], [48].

Our NLP formulation presents several advantages and limitations in pathway optimization. On the negative side, it is clear that verification of the presence or absence of each reaction in the generic topology, or unique identification of its parameters is not possible given the relatively small dataset at hand. Figure 6 shows the un-compartmentalized version of the initial pathway where 116 out of 228 reactions (∼50%) could not be identified if they are present or not given the data at hand (blue lines in Figure 6). This implies that the optimization problem incorporates more parameters than what it is possible to constrain. However, the exhaustive removal of reactions from the PKN, in the case of the medium scale topology, and the adoption of the equivalent classes concept (introduced in [18]) as a compartmentalization scheme, in the case of the large-scale topology, limited the number of redundant/non-identifiable reactions left in the model. Another inherent limitation of the proposed approach is our restriction to connectivity present in the PKN. The formulation we use, by optimizing the values of model parameters (*a* and *p*), minimizes measurement prediction mismatch. Essentially reactions can be removed by setting the gain parameter of the respective reactions to zero, however, there is no support for adding new connections. Thus, the connectivity of proteins in the solution is a subset of the connectivity in the PKN. If the data dictates connectivity that is not supported by the PKN, there will be remaining fitness error in the solution. Even though methods have been developed to address this [51] based on the inference of physical interactions of proteins from the signaling data, adding new connectivity in the PKN can lead to poorly confined solutions and further research is needed to tackle this issue. Another limitation is the single time point measurement of the signaling activity. All the incorporated signaling data from HepG2 cells were obtained from the same time-point (30 min). Consequently, any activity that takes place earlier or later on will not be accounted for. To alleviate this limitation an average “early” time point was employed in the phosphoprotein activity of primary hepatocytes that incorporates the average activity of 5 and 25 minutes [48].The single time point measurements also prevent us from capturing the dynamics of the signaling reactions. Even though a dynamic representation is closer to reality, and can be potentially handled within a logic framework [53], both the experimental cost and the number of parameters required, make it difficult to model large topologies. On the positive side, our approach is a significant advancement of the Boolean Logic that successfully addresses both the protein connectivity and the activity/intensity of reactions in large signaling pathways that –as shown- number ∼120 species and ∼230 reactions.

When compared to Boolean modeling, the proposed approach provides a quantitative view of the signaling pathway, supporting continuous values for the activation of the included species. Moreover, each reaction is modeled via a sigmoid curve (normalized hill function) that more closely replicates its actual mechanics. As a result, the proposed approach gives lower fitness error than the Boolean counterpart. When compared to other fuzzy models, the proposed algorithm performed equally good to previous approaches [33] interrogating the optimization of the medium scale pathway to signaling data. Even though the two procedures follow different workflows, the topology of the solutions is very similar and the goodness of fit is of the same level, whereas CPU times favors the NLP approach (∼60 minutes per run for CellNOpt-cFLagainst ∼15 mins for NLP).

The computational efficiency of the NLP approach allowed the interrogation of large-scale pathways, namely the one introduced in [48]. It performed significantly better than the Boolean approach in terms of goodness of fit, decreasing the fitness error up to 75% (mean fitness error). Although the CPU time was increased, the solution remained computationally feasible.

Overall, the proposed approach addressed successfully the optimization of medium and large-scale signal transduction networks. It allowed the fast optimization of signaling topologies by combining the versatile nature of logic modeling with state of the art optimization algorithms

## Methods

### 1 NLP formulation

The proposed NLP formulation is built based on a pre-existing ILP (Integer Linear Programming) formulation first published in [47] and thus uses the same nomenclature, repeated here for consistency.

#### 1.1 Definitions

A pathway is defined as a set of reactions *i = 1*, …,*n_r_*; and species *j = 1*, …,*n_s_* . Each reaction has three corresponding index sets. Namely the index set of signaling molecules (or reactants) *R_i_* , inhibitors *I_i_* , and “products” *P_i_*. These sets are all subsets of the species index set 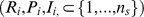; Typically, these subsets have very small cardinality (few species), e.g., | *R_i_* | = 0,1,2 ; | *I_i_* | = 0,1 ; | *P_i_* | = 1,2 ;

| *R_i_* |+| *I_i_* | = 1,2.

A set of in-silico experiments is performed mimicking the conditions of each actual experiment. The experiments are indexed by the superscript *k = 1*, …,*n_e_*. In each experiment a subset of species is introduced to the system and another subset is excluded from the system, in similar fashion to the “actual” experiments where a combination of stimuli and inhibitors are introduced to the cells. The predicted activation value of species *j* in experiment *k* is represented by the constant 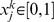. If available, the corresponding measured value is represented by 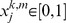. The last group of variables introduced, 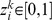, represent the activity of reaction *i* in experiment *k.*

#### 1.2 Objective Function

The objective function to be minimized is



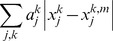
(1)and represents the weighted measurement-prediction mismatch; 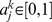
are user- set weights that may favor the fit of specific nodes in the pathway. In the present study, all nodes are considered equally important (have equal weights a_j_^k^).

#### 1.3 Single reactant – single product reactions

Reactions with a single reactant and a single product are modeled using the following transfer function (TF):



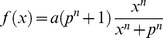
(2)


Equation (2) represents a normalized Hill function. Parameter *p* defines the midpoint of the curve (i.e. the value of *x* for which *f(x)* equals to 0.5), *n* is the Hill coefficient and defines the steepness of the curve whereas *a* is a scaling factor. The activity of reaction *i* in experiment *k* equals to: 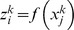, where 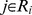. The activation value of the downstream node equals to: 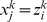, where 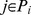. In case species *j* is inhibitory we use: 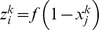, where 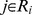.

#### 1.4 Multiple reactants – single product reactions (AND gates)

In case more than one reactants are needed to propagate the signal to the downstream species, the activity of reaction *i* is modeled as a function of the bilinear product of the reacting species:
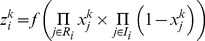
(3)


The activation value of the downstream node equals to: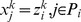. The bilinear product is chosen for satisfying key properties, such as continuity, differentiability and for reproducing the Boolean AND gate for 0 and 1 values of the reacting species.

#### 1.5 Multiple reactions leading to same product (OR gates)

In case more than one reactions lead to the same product, the activation value of the downstream species is given by the following formulation:
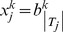
(4)where, 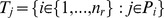
(5)



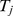
 is the set of all reactions that have species *j* as their product. Let *i_1_, i_2_*, …,*i_|Tj|_* denote the elements of 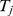. Then, 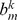
is calculated recursively as:



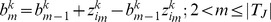
(6)




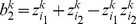
(7)


#### 1.6 Implementation

The goal of the NLP formulation, described above, is the identification of optimal values for *a, p* and *n* parameters of each reaction to minimize the difference between model predictions and experimental data, as captured by the objective function in (1). The NLP was solved through IPOPT under GAMS. Additionally, an interface was developed in BASH scripting language to preprocess the PKN and generate the input files for the NLP algorithm. The DataRail toolbox was employed in MATLAB to handle and plot the dataset [52]. The optimization was run on Dual Quad Core Intel® Xeon® Processors E5530 2.4 GHz, 12 GB, DDR3 RDIMM Memory, 1066 MHz. All results presented in this MS were computed using a single cor.

### 2 Definition of the search space

A systematic definition of the search space is vital for obtaining the best possible solutions within reasonable CPU time. A wider search space accounts for a bigger number of feasible solutions, possibly including some that minimize the objective function, but often increases the CPU time.

The model parameters to be estimated are: *a, p* and *n; a* serves as a scaling factor to limit protein activity in case the reaction appears not to be functional based on the data at hand, and is defined in 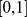; *p* defines the midpoint of the curve (i.e. when *x_j_^k^* equals to 0.5) and can be any real number; *n* can be any positive integer, but here is fixed to 4, since the remaining parameters suffice to fit the data. In the toy model *p* was arbitrarily defined in 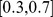. For the medium and large-scale topologies, we test a number of different upper-lower bound pairs, ranging from 0.1 to 2.0, to determine the one for which the algorithm performs best, in terms of goodness of fit, as well as decrease the required CPU time, facilitating the generation of a family of solutions. Goodness of fit is quantified by the mean absolute error (MAE) as calculated by the following formula



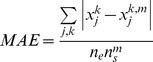
(8)


Results for the medium-scale topology are shown in Figure S1A. The x-axis (0.1→2.0) corresponds to the lower bound of *p* range; y-axis (0.1→2.0) corresponds to the upper bound; while the z-axis corresponds to the MAE of the solution. Figure S1A shows that the quality of the solution mostly depends on the lower bound and less on the upper bound of *p.* In Figure S1B the corresponding CPU time is shown. As expected widening the range of *p* drastically increases the CPU time, since the search space becomes bigger. Based on these graphs the bounds of choice for *p* is 0.1 → 0.4375, since they provide both an excellent fit and low CPU time.

### 3 Generation of a family of solutions

Instead of collecting a single solution that minimizes the objective function in (1), we collect a family of 500 near optimal solutions to account for slightly suboptimal pathways that may bare strong biological significance, and avoid as much as possible terminating with a significantly suboptimal local minimum.

The proposed NLP approach optimizes the values of *a* and *p* to minimize the measurement – prediction mismatch as shown in equation (1). However, as long as the optimizer used is local, there is no guarantee that the obtained solution is a global minimum of (1). Moreover, there might be more than one solution (with different values for *a* and *p*), scoring the same (optimal) goodness of fit, which should be taken in consideration when biological insight about the interrogated system is to be extracted. Therefore, a large number of runs is performed each one starting from different (random) initial guesses, to obtain a family of near optimal solutions. Figure S3A shows the MAE of 500 solutions, obtained from equal runs of the proposed NLP approach each one starting from a different initial guess for the parameters *a* and *p.* Most of the runs resulted in solutions with very similar (±3%) MAEs. This indicates that although the IPOPT optimizer, used herein, is not global, it furnishes near-optimal solution points independently on the initial guess. In Figure 3C, an “average” pathway for these 500 runs is illustrated. The opacity of each of these edges corresponds to the average activity of the respective reactions over the 500 runs. For a discussion on the optimum size of the family of solutions see Supporting Information 1.

### 4 Removing conflicting and redundant reactions from the PKN

Optimization of the PKN to the data at hand results in a set of values for the model parameters (*a* and *p*) that minimize the measurement prediction mismatch, as defined in equation (1). Subsequently, we iteratively remove reactions from the PKN (every time a reaction is removed we re-optimize the PKN) while monitoring the fitness error to identify all reactions that are not vital in fitting the signaling dataset, either because they directly contradict the data, or because they are non-identifiable. Non-identifiable reactions are those whose presence in the model cannot be validated nor disproven based on the data at hand. This may occur when signal transduction from a cytokine to a measured protein can be achieved by a number of different pathways, and there is no definite way to identify which one is really functional. Consequently, removing a non-identifiable reaction from the PKN has no effect on the fitness error.

In an attempt to remove conflicting reactions and tackle over-parameterization, we gradually remove reactions from the PKN until the fitness error starts increasing (i.e., the algorithm can no longer fit the dataset at hand). At that point there are no more conflicting or non-identifiable reactions left in the model, but all of the remaining ones are vital for fitting the data. At every iteration, the reaction with the lowest activity is removed (variable *z_i_^k^* in the formulation). The activity of each reaction mostly depends on the parameter *a* (gain) of the reaction and directly correlates to the “amount of signal” propagating downstream. In this manner, the least significant reaction is removed at every iteration. Even though the sequence reactions are removed by will affect the obtained solution (i.e., the solution is not unique), it is guaranteed to be optimal since only conflicting/non-identifiable reactions are removed and key property of these reactions is that their removal does not affect the fitness error of the solution.

Results are illustrated in Figure S2. Figure S2 shows how the algorithm performs when reactions of the PKN are removed in order of increasing significance. The x-axis corresponds to the number of removed reactions, while the y-axis corresponds to the MAE of the solution. As illustrated in Figure S2, up to 10 reactions can be removed (20% of the initial topology) without affecting the goodness of fit of the solution. More than that, vital reactions are missing and the MAE increases significantly. Small fluctuations in the figure are attributed to variations of the fitness error of the solutions (±3%). Figure 3C, 3D shows the solution after removing conflicting and non-identifiable/redundant reactions.The above-mentioned procedure results in the identification of one of possibly many optimal and identifiable solutions, the superposition of which is the family of solutions as defined in paragraph 6.3.

### 5 Compartmentalization of the large-scale topology

Before optimizing the large-scale model, the PKN is compartmentalized by grouping together nodes that share identical response under all experimental conditions, to reduce the parameter space.

In similar fashion to the medium-scale model in Figure 3, the large-scale pathway in Figure 6 also includes a number of non-identifiable reactions, in the sense that signal transduction from a cytokine to a measured protein can be achieved by a number of different pathways and there is no definite way to identify which one is truly functional. In pathways of this size, however, is not efficient to exhaustively remove reactions until the optimizer can no longer fit the data at hand. Instead we propose an alternative method for reducing the parameter space. We propose a compartmentalization scheme, based on the “equivalent classes” concept introduced in [18], for “grouping” nodes that share identical responses under all experimental conditions; thus resulting in an equivalent (compartmentalized) model where nodes have been replaced with their respective compartments, and reactions between nodes are now reactions between compartments. In more detail, we define a *compartment (C) as* every set of *non-measured* species (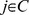), such that 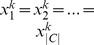
for every 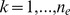. Where

- *k = 1*, …,*n_e_*, is the set of experiments.

- *x_j_^k^* is the predicted value of species *j* in experiment *k*.

In this case study, we simulate the pathway running the NLP formulation under all experimental conditions present in the signaling dataset with nominal values for all parameters; subsequently, we format the simulation results in a 2d matrix, rows corresponding to the nodes in the pathway and columns corresponding to the different experimental conditions; we identify the nodes that share the same response under all conditions (i.e., identify replicate lines) and group them together in compartments; we replace every node in the PKN with its corresponding compartment and remove replicate reactions. This procedure is implemented using BASH. Since the nodes in a compartment share identical responses under all experimental conditions, their connectivity inside the compartment cannot, in principle, be interrogated based on the data at hand. Thus, it is purposeful to group these nodes together and update the PKN replacing nodes with the compartments they belong into. By doing so, we drastically decrease the parameters space.

### Application of the compartmentalization scheme to an illustrative example

To better illustrate how the proposed compartmentalization scheme works to simplify the interrogated model, we construct the example model of Figure S4A. Node “A” serves as input to the pathway (stimuli), and activates nodes B1, B2; these interact with each other and finally activate node “C” that serves as a readout (signal). The proposed scheme groups B1-B2 into “Cmp” and simplifies the model as illustrated in Figure S4B. If data dictates: *A = 1;C = 1*, then reactions A→Cmp and Cmp→C are conserved. Else if *A = 1;C = 0*, then at least one of the above mentioned reactions have to be removed.

Figure S4C, S4D demonstrate how the compartmentalization scheme can be too restrictive and may decrease the quality of the solution. In Figure S4C input nodes A1, A2 are connected to latent nodes B1 and B2; B1 activates C1 and B2 activates C2. After the compartmentalization procedure, B1 and B2 are replaced with compartment “Cmp” that activates C1 and C2 (Figure S4D). In the case where C1 is activated by A1, and C2 by A2; then either C1, or C2 will be misfitted in the compartmentalized model, since differential activation of C1 and C2 is possible only if either CMP→C1, or CMP→C2 are removed from the pathway. However, if either one of the two reactions are removed, then the respective signal (C1 or C2) will remain inactive under all conditions, thus misfitting the data. If no compartmentalization is performed, then the pathway can be optimized by removing (or decreasing the activity) of A1→B2 and A2→B1. This increase in fitness error caused by the compartmentalization procedure implies that grouping nodes B1 and B2 in the compartment Cmp should not have taken place if data were to fit perfectly. Cases like this may arise when limited experimental conditions are available, since it is more likely for nodes to be grouped together. E.g. If only one condition is available, then all nodes will be grouped in a single compartment. In such cases compartmentalization of the PKN is not recommended. In all cases the solution should be manually inspected to ensure that the remaining fitness error is not caused by the aggression of the compartmentalization scheme.

## Supporting Information

Figure S1 Systematic selection of the lower and upper bounds of *p* parameters. (A) Mean Absolute Error (MAE) as a function of the lower and upper bounds of *p* parameters of each reaction. The x-axis (0.1→2.0) corresponds to the lower bound of *p* range; y-axis (0.1→2.0) corresponds to the upper bound; while the z-axis corresponds to the MAE of the solution. The figure shows that MAE is mostly affected by the lower bound of *p*, smaller values of the lower bound lead to a better fit of the signaling data. (B) CPU time as a function of the lower and upper bounds of *p* parameters. CPU time is mostly affected by the lower bound of *p.* smaller values of the lower bound lead to increased CPU time.
(PDF)

Figure S2 Addressing over-parameterization (medium scale pathway). Reactions are exhaustively removed from the PKN in order of increasing activity, and the fitness error is monitored. The x-axis shows the number of reactions excluded; the y-axis shows the Mean Absolute Error of the solution. The figure shows the dependency of the MAE from the subset of excluded reactions. Up to 10 reactions can be removed from the PKN without affecting the MAE of the solution (arrow A), implying these 10 reactions are not vital in fitting the signaling data (redundant reactions). Beyond this point vital reactions are removed, the optimization algorithm can no longer fit the data at hand and the fitness error increases drastically.This is where the final (optimal and identifiable) solution is obtained. Small fluctuations in the figure are attributed to variations of the fitness error of the solutions (±3%).
(PDF)

Figure S3 Generation of a family of solutions – medium-scale pathway. (A) The MAEs of a family of 500 near optimal solutions. The x-axis corresponds to the different runs; the y-axis corresponds to the MAE of the solution. (B) Standard deviation of the MAEs in a family of solutions as a function of the family's size. The x-axis represents the size of the family of solutions; y-axis represents the standard deviation of the solutions. The bigger the size of the family of solutions the smaller the standard deviation of the solutions becomes, indicating decreased sample variability. Optimum size would be around 150-200 solutions where the standard deviation has dropped close to its final value.
(PDF)

Figure S4 Compartmentalization of illustrative example models. The compartmentalization of two example models is featured. (A) example model with a single input (green node) single output (grey node) and 2 latent nodes (white nodes). (B) Compartmentalized version of the example model in (A). The two latent nodes are grouped in compartment Cmp. (C) example model with two inputs, two outputs and two latent nodes. (D) Compartmentalized version of the example model in (C). The proposed compartmentalization scheme is over-aggressive decreasing the quality of the solution in case the two measured proteins have different response under A1 and A2.
(PDF)

Figure S5 Generation of a family of solutions – large-scale pathway. The MAEs of a family of 170 near optimal solutions are illustrated. The x-axis corresponds to the different runs; the y-axis corresponds to the MAE of the solution. Most of the solutions share the same ,optimal, goodness of fit ensuring the algorithm is not trapped in local minima.
(PDF)

Supporting Information S1 1) An alternative Mixed Integer Non Linear Programming (MINLP) formulation is presented. The MINLP formulation not only solves for the reaction parameters a, p and n, but also interrogates the presence or absence of each reaction by introducing a set of binary variables y, where y = 1 if reaction is present in the optimized solution, or y = 0 otherwise. 2) Optimum size for the family of solutions. The optimum size for the family of solutions is addressed. Instead of collecting a single solution that minimizes the objective function, we collect a number of near optimal solutions to account for slightly suboptimal pathways that may bare strong biological significance, and avoid as much as possible terminating with a significantly suboptimal local minimum. 3) Comparison with the compression scheme implemented in CellNOpt. The proposed compartmentalization scheme is compared against the compression algorithm implemented in CellNOpt [2].
(PDF)
