# Supplementary figures and images for: Non Linear Programming (NLP) Formulation for Quantitative Modeling of Protein Signal Transduction Pathways
Source: PLoS One. 2012 Nov 30;7(11):e50085. doi: 10.1371/journal.pone.0050085 (PMC3511450; doi:10.1371/journal.pone.0050085)

Figure S1

# Systematic selection of the lower and upper bounds of p parameters

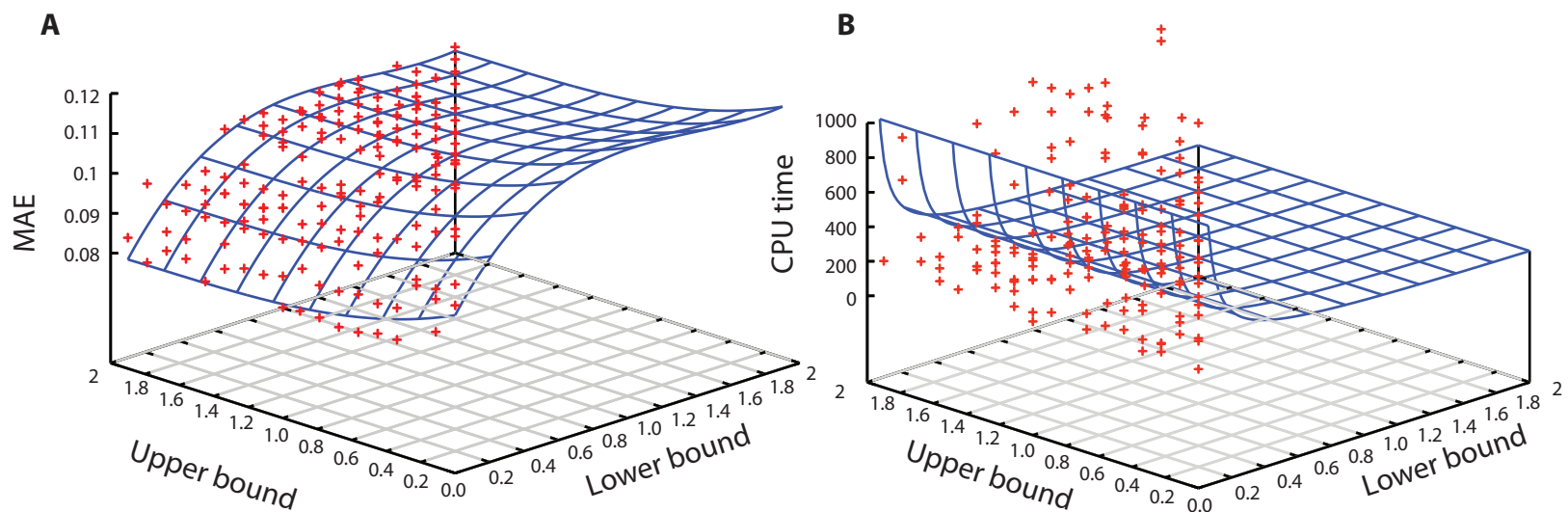

Supplement: Figure S1 — Systematic selection of the lower and upper bounds of p parameters. (A) Mean Absolute Error (MAE) as a function of the lower and upper bounds of p parameters of each reaction. The x-axis (0.1→2.0) corresponds to the lower bound of p range; y-axis (0.1→2.0) corresponds to the upper bound; while the z-axis corresponds to the MAE of the solution. The figure shows that MAE is mostly affected by the lower bound of p, smaller values of the lower bound lead to a better fit of the signaling data. (B) CPU time as a function of the lower and upper bounds of p parameters. CPU time is mostly affected by the lower bound of p. smaller values of the lower bound lead to increased CPU time. (PDF) [file pone.0050085.s001.pdf]

Figure S2

### Removal of redundant reactions

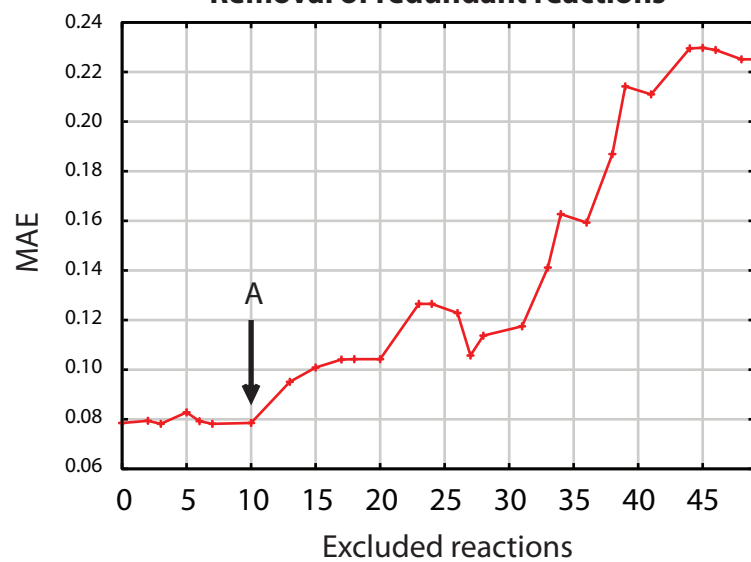

Supplement: Figure S2 — Addressing over-parameterization (medium scale pathway). Reactions are exhaustively removed from the PKN in order of increasing activity, and the fitness error is monitored. The x-axis shows the number of reactions excluded; the y-axis shows the Mean Absolute Error of the solution. The figure shows the dependency of the MAE from the subset of excluded reactions. Up to 10 reactions can be removed from the PKN without affecting the MAE of the solution (arrow A), implying these 10 reactions are not vital in fitting the signaling data (redundant reactions). Beyond this point vital reactions are removed, the optimization algorithm can no longer fit the data at hand and the fitness error increases drastically.This is where the final (optimal and identifiable) solution is obtained. Small fluctuations in the figure are attributed to variations of the fitness error of the solutions (±3%). (PDF) [file pone.0050085.s002.pdf]

Figure S3

### Generation of a family of solutions - medium scale pathway

**A**

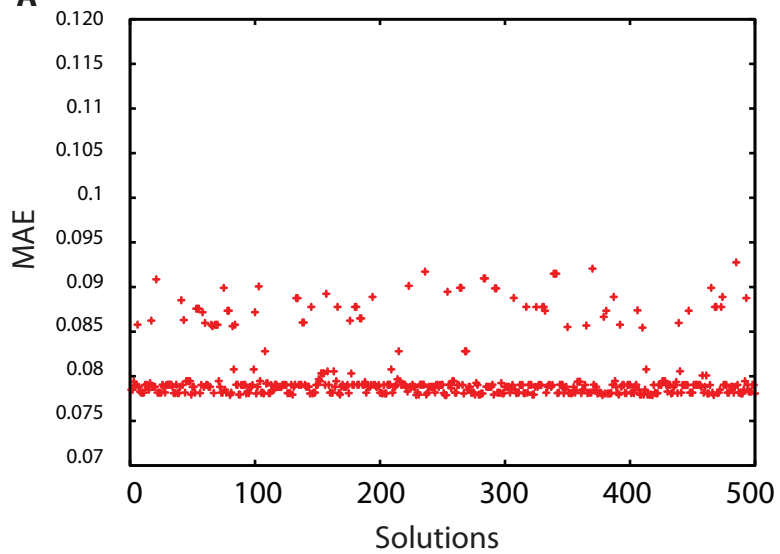

**B**

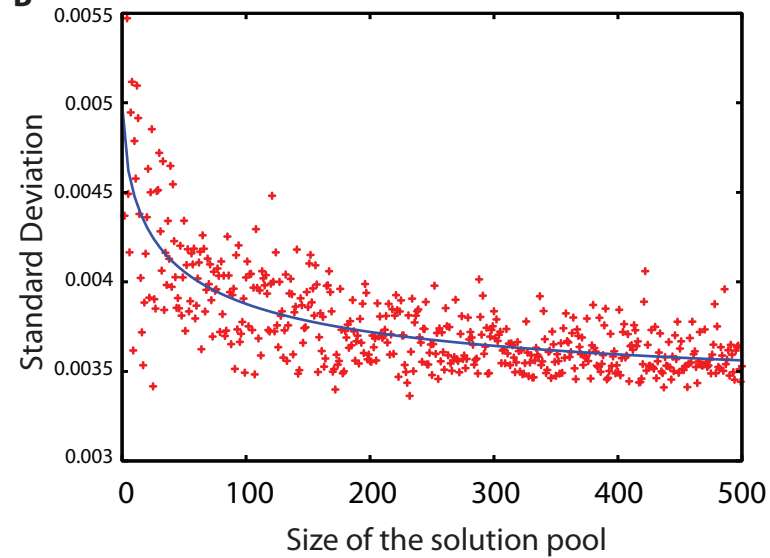

Supplement: Figure S3 — Generation of a family of solutions – medium-scale pathway. (A) The MAEs of a family of 500 near optimal solutions. The x-axis corresponds to the different runs; the y-axis corresponds to the MAE of the solution. (B) Standard deviation of the MAEs in a family of solutions as a function of the family's size. The x-axis represents the size of the family of solutions; y-axis represents the standard deviation of the solutions. The bigger the size of the family of solutions the smaller the standard deviation of the solutions becomes, indicating decreased sample variability. Optimum size would be around 150-200 solutions where the standard deviation has dropped close to its final value. (PDF) [file pone.0050085.s003.pdf]

Figure S4

Compartmentalization of illustrative example models

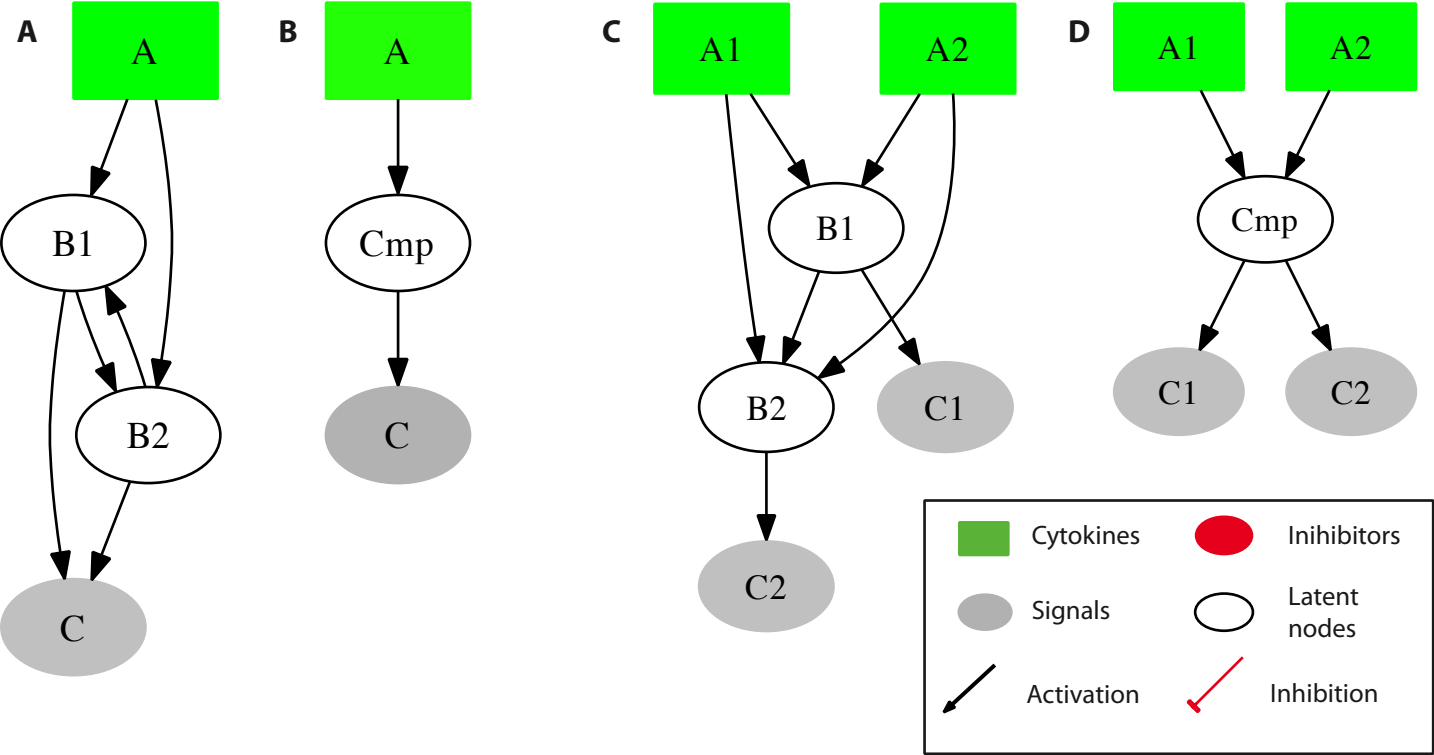

Supplement: Figure S4 — Compartmentalization of illustrative example models. The compartmentalization of two example models is featured. (A) example model with a single input (green node) single output (grey node) and 2 latent nodes (white nodes). (B) Compartmentalized version of the example model in (A). The two latent nodes are grouped in compartment Cmp. (C) example model with two inputs, two outputs and two latent nodes. (D) Compartmentalized version of the example model in (C). The proposed compartmentalization scheme is over-aggressive decreasing the quality of the solution in case the two measured proteins have different response under A1 and A2. (PDF) [file pone.0050085.s004.pdf]

Figure S5

### Generation of a family of solutions - large scale pathway

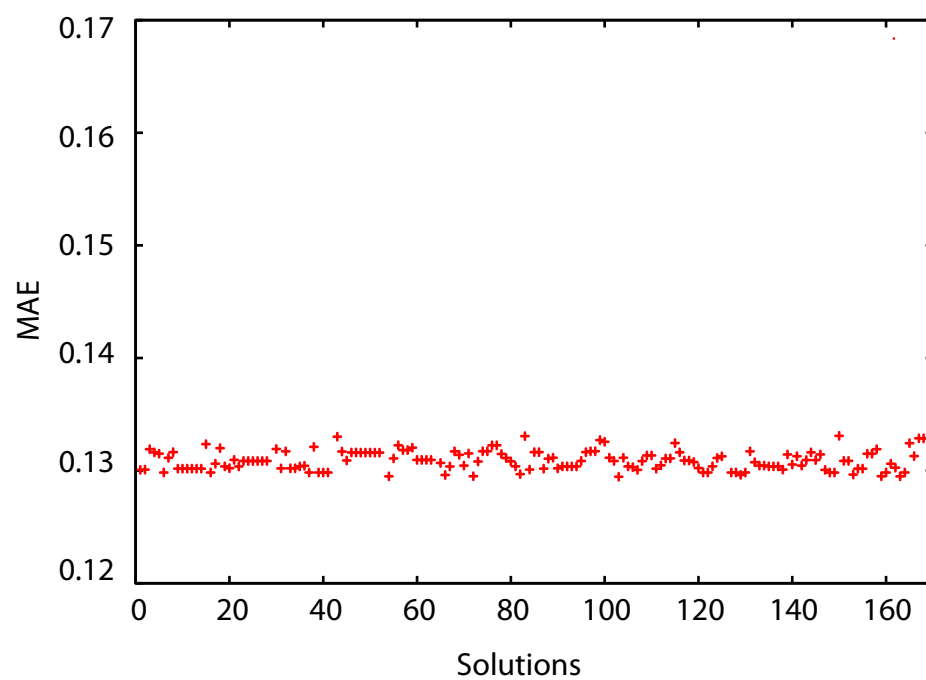

Supplement: Figure S5 — Generation of a family of solutions – large-scale pathway. The MAEs of a family of 170 near optimal solutions are illustrated. The x-axis corresponds to the different runs; the y-axis corresponds to the MAE of the solution. Most of the solutions share the same ,optimal, goodness of fit ensuring the algorithm is not trapped in local minima. (PDF) [file pone.0050085.s005.pdf]
