# Supplementary material for: Non Linear Programming (NLP) Formulation for Quantitative Modeling of Protein Signal Transduction Pathways
Source: PLoS One. 2012 Nov 30;7(11):e50085. doi: 10.1371/journal.pone.0050085 (PMC3511450; doi:10.1371/journal.pone.0050085)
Supplement: Supporting Information S1 — 1) An alternative Mixed Integer Non Linear Programming (MINLP) formulation is presented. The MINLP formulation not only solves for the reaction parameters a, p and n, but also interrogates the presence or absence of each reaction by introducing a set of binary variables y, where y = 1 if reaction is present in the optimized solution, or y = 0 otherwise. 2) Optimum size for the family of solutions. The optimum size for the family of solutions is addressed. Instead of collecting a single solution that minimizes the objective function, we collect a number of near optimal solutions to account for slightly suboptimal pathways that may bare strong biological significance, and avoid as much as possible terminating with a significantly suboptimal local minimum. 3) Comparison with the compression scheme implemented in CellNOpt. The proposed compartmentalization scheme is compared against the compression algorithm implemented in CellNOpt [2]. (PDF) [file pone.0050085.s006.pdf]

***Supplementary material***

## *1. An alternative Mixed Integer Non Linear Programming formulation*

Apart from the Non Linear Programming (NLP) formulation described in the main text of this paper, we derived a Mixed Integer Non Linear Programming (MINLP) formulation to address the optimization ofthe Prior Knowledge Network (PKN) to signaling data. The MINLP formulation not only solves for the reaction parameters (*a, p* and *n,* see Methods section §5.1) but also interrogates the presence or absence of each reaction by introducing a set of binary variables $y_i \in \{0, 1\}$, where $i=1,\ldots,n_r$ is the set of reactions, $y_i=0$ implies reaction *i* is absent, $y_i=1$ implies reaction *i* is present (see also [1]). Even though the MINLP is capable of optimizing the connectivity of the proteins in the signaling network together with the mechanics of each reaction, it introduces an additional parameter for each reaction, increasing the complexity of the optimization problem and subsequently the CPU time.

Apart from the constraints (3)-(7) in materials and methods section (5.1) the following inequality must be incorporated.

$$z_i^k \le y_i \ ; \ i = 1,\ldots,n_r \ ; \ k = 1,\ldots,n_e \qquad (9)$$

Constraint (9) implies that reaction *i* can be active only if it is present.Thus, constraint (3), in the case of AND gates, becomes:

$$z_i^k = y_i \cdot f\left(\prod_{j \in R_i} x_j^k \times \prod_{j \in J_i} \left(1 - x_j^k\right)\right) \qquad (3')$$

In the case of OR gates the activation value of the downstream species is given by:

$$x_j^k = b_{|T_j|}^k \qquad (4)$$

where, $T_j = \{i \in \{1,\ldots,n_r\} : j \in P_i\}$ (5)

$T_j$ is the set of all reactions that have species *j* as their product. Let $i_1, i_2, \ldots, i_{|T_j|}$ denote the elements of $T_j$. Then, $b_m^k$ is calculated recursively as:

$$b_m^k = b_{m-1}^k + z_{i_m}^k - b_{m-1}^k \ z_{i_m}^k \ ; 2 < m \le |T_J| \qquad (6)$$

$$b_2^k = z_{i_1}^k + z_{i_2}^k - z_{i_1}^k \ z_{i_2}^k \qquad (7)$$

where, $z_i^k = y_i \cdot f\left(x_{j \in R_i}^k\right)$.

The $y_i$ variables allow for the explicit removal of reactions that appear to contradict the data at hand, in contrast to the proposed NLP formulation where reactions are removed implicitly by setting the *a* parameters to 0. Although the MINLP approach successfully optimized small and medium scale topologies, the

proposed NLP approach performed significantly better in terms of goodness of fit to the data and CPU time, thus it is the method of choice for the analysis presented in this paper. Both the NLP and the MINLP variants are susceptible to local minima of the objective function, thus a family of solutions must be obtained to guarantee that all biologically significant solutions have been accounted for.

### *2. Optimum size for the family of solutions*

The proposed NLP formulation aims at optimizing the values of *a, p* and *n* parameters in a way that the fitness error to the training data is minimized. Instead of collecting a single solution that minimizes the objective function, we collect a number of near optimal solutions to account for slightly suboptimal pathways that may bare strong biological significance, and avoid as much as possible terminating with a significantly suboptimal local minimum. In Supplementary Figure 3A, the fitness error of a family of 500 solutions is illustrated.

In Supplementary Figure 3B the optimum size for the family of solutions is addressed. We construct 500 solution pools, each one numbering from 1 to 500 solutions. Then the mean and standard deviation of the fitness error of each solution pool is evaluated. Supplementary Figure 3B shows that small sizes of the family of solutions leads to increased standard deviation since the sample demonstrates greater variability. Optimum size would be around 150-200 solutions where the standard deviation has dropped close to its final value.

### *3. Compartmentalization ofillustrative example models*

To better illustrate how the proposed compartmentalization scheme works to simplify the interrogated model, we construct the example model of supplementary Figure 4A. Node "A" serves as input to the pathway (stimuli), and activates nodes B1, B2; these interact with each other and finally activate node "C" that serves as a readout (signal).The proposed scheme groups B1-B2 into "Cmp" and simplifies the model as illustrated in supplementary Figure 4B. If data dictates: *A=1;C=1,* then reactions A→Cmp and Cmp→C are conserved. Else if *A=1;C=0,* then at least one of the above mentioned reactions have to be removed.

Another preprocessing algorithm is the one introduced in [2] and used within the CellNOpt-cFL framework; briefly 2 rules were defined for simplifying the PKN:

i) Remove all nodes that are either non-observable or non-controllable and reactions to/from these nodes. Non-observable are nodes downstream of which there are no signals. Non-controllable are nodes that are downstream of no stimuli.

ii) Compress linear chains of reactions such as A->B->C, to A->C. Where the intermediate nodes are not signals.

Eventhough this collection of rules is effective against a wide range of topologies it cannot handlenodes with multiple inputs and multiple outputs, such as the topology

of this example model. Compression of the example model under CellNOpt will not simplify the structure of the pathway.

Supplementary Figure 4C, 4D demonstrate how the compartmentalization scheme can be too restrictive and may decrease the quality of the solution. In Supplementary Figure 4C input nodes A1, A2 are connected to latent nodes B1 and B2; B1 activates C1 and B2 activates C2.After the compartmentalization procedure, B1 and B2 are replaced with compartment “Cmp” that activates C1 and C2 (Supplementary Figure 4D). In the case where C1 is activated by A1, and C2 by A2; then either C1, or C2 will be misfitted in the compartmentalized model, since differential activation of C1 and C2 is possible only if either CMP→C1, or CMP→C2 are removed from the pathway. However, if either one of the two reactions are removed, then the respective signal (C1 or C2) will remain inactive under all conditions, thus misfitting the data. If no compartmentalization is performed then the pathway can be optimized by removing (or decreasing the activity) of A1→B2 and A2→B1.This increase in fitness error caused by the compartmentalization procedure implies that grouping nodes B1 and B2 in the compartment Cmp should not have taken place if data were to fit perfectly.Cases like this may arise when limited experimental conditions are available, since it is more likely for nodes to be grouped together. E.g. If only one condition is available, then all nodes will be grouped in a single compartment. In such cases compartmentalization of the PKN is not recommended. In all cases the solution should be manually inspected to ensure that the remaining fitness error is not caused by the aggression of the compartmentalization scheme.

## *References*


1. Mitsos A, Melas IN, Siminelakis P, Chairakaki AD, Saez-Rodriguez J, et al. (2009) Identifying Drug Effects via Pathway Alterations using an Integer Linear Programming Optimization Formulation on Phosphoproteomic Data. Plos Computational Biology 5: -.
2. Saez-Rodriguez J, Alexopoulos LG, Epperlein J, Samaga R, Lauffenburger DA, et al. (2009) Discrete logic modelling as a means to link protein signalling networks with functional analysis of mammalian signal transduction. Molecular Systems Biology 5: -.
